# Reliability of Machine and Human Examiners for Detection of Laryngeal Penetration or Aspiration in Videofluoroscopic Swallowing Studies

**DOI:** 10.3390/jcm10122681

**Published:** 2021-06-18

**Authors:** Yuna Kim, Hyun-Il Kim, Geun Seok Park, Seo Young Kim, Sang-Il Choi, Seong Jae Lee

**Affiliations:** 1 Department of Rehabilitation Medicine, Dankook University Hospital, Cheonan 31116, Korea; kimyuna727@dkuh.co.kr (Y.K.); geunpark@dkuh.co.kr (G.S.P.); juliet8383@naver.com (S.Y.K.); 2 Department of Computer Science and Engineering, Dankook University, Yongin 16890, Korea; gusdlf93@naver.com; 3 Department of Computer Engineering, Dankook University, Yongin 16890, Korea; 4 Department of Rehabilitation Medicine, College of Medicine, Dankook University, Cheonan 31116, Korea

**Keywords:** dysphagia, swallowing, laryngeal penetration or aspiration, deglutition, reliability, videofluoroscopic swallowing study, deep learning, machine learning

## Abstract

Computer-assisted analysis is expected to improve the reliability of videofluoroscopic swallowing studies (VFSSs), but its usefulness is limited. Previously, we proposed a deep learning model that can detect laryngeal penetration or aspiration fully automatically in VFSS video images, but the evidence for its reliability was insufficient. This study aims to compare the intra- and inter-rater reliability of the computer model and human raters. The test dataset consisted of 173 video files from which the existence of laryngeal penetration or aspiration was judged by the computer and three physicians in two sessions separated by a one-month interval. Intra- and inter-rater reliability were calculated using Cohen’s kappa coefficient, the positive reliability ratio (PRR) and the negative reliability ratio (NRR). Intrarater reliability was almost perfect for the computer and two experienced physicians. Interrater reliability was moderate to substantial between the model and each human rater and between the human raters. The average PRR and NRR between the model and the human raters were similar to those between the human raters. The results demonstrate that the deep learning model can detect laryngeal penetration or aspiration from VFSS video as reliably as human examiners.

## 1. Introduction

The videofluoroscopic swallowing study (VFSS) is currently regarded as the gold standard method for evaluating swallowing function because it allows real-time visualization of bolus movement along with the dynamics of anatomical structures associated with the swallowing process [1,2]. A VFSS makes it possible to detect the presence and timing of laryngeal penetration or aspiration and helps to identify its physiological mechanisms [2,3,4].

The videofluoroscopic images are recorded while the patients swallow boluses mixed with contrast, and physicians or speech–language pathologists analyze the recorded videos [2]. VFSS analysis depends on the subjective visual judgment of the reviewers and is inevitably susceptible to human bias [5,6,7]. Human examiners usually have the burden of reviewing the images dozens of times for one patient because the swallowing process is repeated 10 to 15 times per test and repeated replay is required due to the fast and complex nature of swallowing. Consequently, it is difficult to avoid human error due to the fatigue that results from high concentration and repetitive examination. Because of this vulnerability to human error, the reported reliability of VFSS analysis is not excellent; wide variation is present in both intra- and inter-rater agreement (intrarater к = 0.530~1.00, interrater к = 0.269~0.700) [5,6,7,8,9].

As an alternative to overcome the limitations of human reading, recent studies have attempted to develop computer-assisted analysis [10,11,12,13,14,15]. Aung et al. suggested that automated reading enables more objective and immediate analysis with a quantifiable level of accuracy, eliminates the need for high levels of training for analysis and reporting, and provides a platform for larger-scale screening of populations with dysphagia [10]. Computer-assisted analysis typically tracks anatomical landmarks automatically after they are demarcated by humans in the first few frames of the videos [10,11,12,13,14,15]. However, its clinical usefulness has been limited because most of the models use obsolete semiautomated tracking and segmentation algorithms that require manual demarcation of anatomical landmarks.

Recently, deep learning technology has increased the accuracy of image classification to a level exceeding that of human eyes and is expected to reduce error in reading medical images [16,17,18,19]. In a previous study, we developed and proposed a model capable of detecting laryngeal penetration or aspiration from VFSS images in a fully automated manner without any human intervention by applying deep learning algorithms [20]. The model showed an overall accuracy of 97.2% in classifying image frames and 93.2% in classifying video files in which laryngeal penetration or aspiration was evident, exceeding the accuracy of previous semiautomated computer-assisted analysis. The results showed the potential value of the model for clinical practice in many respects, but the evidence for its reliability still seems to be insufficient.

This study aims to examine and compare the intra- and inter-rater reliability of our deep learning model and human examiners for the detection of laryngeal penetration or aspiration from VFSS images. We anticipate that the results of this study may provide further evidence to support the clinical application of deep learning technology in VFSS analysis, although dichotomous results of whether penetration/aspiration was detected or not on VFSS does not always represent the degree of pathology in the swallowing mechanism.

## 2. Materials and Methods

### 2.1. Dataset

We collected a total of 205 VFSS video files from 49 patients, aiming for an even distribution of attributes including gender, age, viscosity of diet and degree of laryngeal penetration or aspiration. Presence of the penetration or aspiration was determined using the PAS (Penetration/Aspiration Scale) [21] and videos scored as PAS 2 or higher were included. The video files were selected from the database of Dankook University Hospital, which contains the videos of VFSSs conducted between January 2015 and June 2020. The VFSS was performed according to the protocol described by Logemann [22] with minor modifications. Briefly, video images were acquired via lateral projection at a speed of 30 fps (frames per second) while the seated patients swallowed boluses of various consistencies mixed with contrast medium; the videos were stored digitally. The types of boluses swallowed were as follows: 3 mL of thick liquid (water-soluble barium sulfate diluted to 70%); 3 mL of rice porridge; 3 mL of curd-type yogurt; 3 mL of thin liquid (water-soluble barium sulfate diluted to 35%) from a spoon; or 5 mL of thin liquid from a cup. The video files were selected by an investigator who had more than two years of experience in analysis of VFSS. Every effort was made to select videos in which the presence or absence was evident. The video files were edited to contain only one swallowing event. Each swallowing was defined as the process from the backward movement of bolus in oral cavity to the returning of larynx to original position. A little space was also put on the front and back of the swallowing event to include the whole swallowing event. When the bolus was not fully swallowed in first attempt, subsequent swallows were also included until the bolus was completely swallowed. The videos were not included if they showed remaining of the bolus aspirated from previous swallow in the larynx. Among those files, 32 were excluded due to poor image quality. Ultimately, 173 video files from 42 patients were included in the VFSS dataset; the distribution of their attributes is shown in Table 1. The shortest video lasted 4 s, and the longest video lasted 240 s. The depth of penetration/aspiration was categorized as shallow (PAS 2 or 3), deep (PAS 4 or 5) and aspiration (PAS 6 or higher) and their distribution is shown in Table 1. The proportion of presence and depth was set to equal the overall distribution in database of authors’ institution.

### 2.2. Analysis of VFSS

#### 2.2.1. Machine Reading

**Figure 1:**
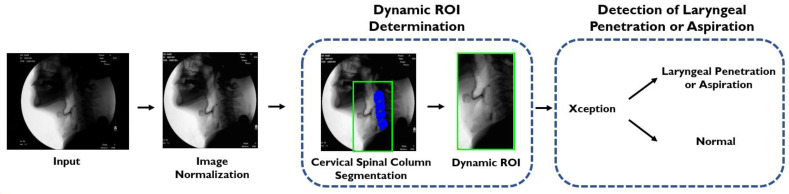
The same deep learning model we proposed in our previous study [20] is used in this study. After normalization of the input images, a dynamic ROI is defined with reference to the cervical spinal column segmented by U-net. The presence of laryngeal penetration or aspiration in the ROI can be identified by the Xception module.

**Figure 2:**
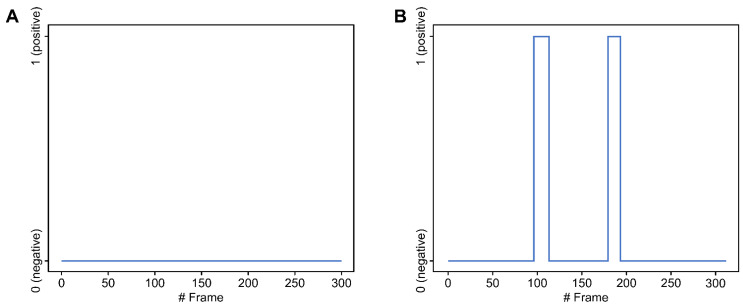
Example output of the deep learning model represented as histograms: (**A**) No laryngeal penetration or aspiration was detected in any frame of the video. (**B**) Laryngeal penetration or aspiration occurred in approximately the 100th to 115th frames and the 180th to 200th frames of the video.

The video files were examined for the presence of laryngeal penetration or aspiration using the computer model described in a previous study [20]. In summary, the model consisted of three phases: (1) image normalization, (2) dynamic ROI (region of interest) determination, and (3) detection of laryngeal penetration or aspiration (Figure 1). After the input images were normalized using CLAHE (contrast-limited adaptive histogram equalization) [23], an ROI was defined with reference to the cervical spinal column segmented using U-net. The ROI was set to include the larynx, the cervical spine, and adjacent areas. Noise from the movement of head and neck could be minimized by setting the ROI to move dynamically with the cervical spines. Within the ROI, the presence of laryngeal penetration or aspiration was classified by the deep learning network trained with the Xception module [24]. The output was reported and displayed in the form of histograms as shown in Figure 2. The classification and reporting process was conducted in a fully automated manner without any human intervention except for inputting the image data. Display of at least one peak was considered “positive” result.

#### 2.2.2. Human Reading

The human raters were three physicians: “Human 1”, with more than 20 years of experience in VFSS analysis; “Human 2”, with 10 years; and “Human 3”, the novice with 1 year. Working in separate locations, the three human examiners judged the existence of laryngeal penetration or aspiration, regardless of severity or depth, in the same video files. When multiple swallowing attempts were included in the video clip, the result was rated as “positive” if any one of the attempts shows penetration/aspiration. Discussion was not allowed, and no information about the subjects in the videos (including gender, age, and medical history) or the viscosity of the bolus was given to the raters.

### 2.3. Analysis of Intra- and Inter-Rater Reliability

#### 2.3.1. Intrarater Reliability

Trials were conducted in two sessions, separated by four weeks, to calculate the intrarater reliability of machine and human reading. In both sessions, the presence or absence of laryngeal penetration or aspiration was judged by three human raters and the deep learning model. In the second session, 173 video files were reordered and randomly assigned to the raters by an investigator who was blinded to the results of the first session. The results were collected from the three human raters and the model in both sessions, and Cohen’s kappa coefficient was calculated. However, the meaning of epidemiological statistics derived in this way can be limited because there is no absolute gold standard for VFSS analysis. Therefore, we used the positive reliability ratio (PRR) and negative reliability ratio (NRR), as suggested by Kuhlemeier et al. [8]. In the absence of a gold standard, PRR and NRR can provide statistics about the agreement between session results from the same interpreter [8]. According to the definition of Kuhlemeier et al. [8], we calculated the PRR as the percentage of cases a given rater judged abnormal in the first session that he or she also judged abnormal in the second session. The NRR was calculated in the same way for normal ratings.

Therefore, the PRR and NRR were calculated by the following formulas:

PRR = Abn(1 and 2)/Abn(1), where Abn(1 and 2) = number rated abnormal in both the first and second sessions and Abn(1) = number rated abnormal in the first session.

NRR = Normal(1 and 2)/Normal(1), where Normal(1 and 2) = number rated normal in both the first and second sessions and Normal(1) = number rated normal in the first session.

#### 2.3.2. Interrater Reliability

The interrater reliability was verified in the same way as the intrarater reliability. As with the intrarater reliability, the interrater PRR and NRR were defined according to the definition by Kuhlemeier et al. [8]. PRR and NRR were calculated between each possible combination of human raters and machine, not between sessions. For interrater reliability, PRR denoted the percentage of cases judged abnormal (i.e., having laryngeal penetration or aspiration) by rater “A” that were also judged abnormal by rater “B”. In the same way, NRR was calculated based on the cases judged to be normal.

Thus, interrater PRR and NRR were calculated by the following formulas:

PRR = Abn(A and B)/Abn(A), where Abn(A and B) = number rated abnormal by both “A” and “B” and Abn(A) = number rated abnormal by “A”.

NRR = Normal(A and B)/Normal(A), where Normal(A and B) = number rated normal by both “A” and “B” and Normal(A) = number rated normal by “A”.

All statistical analysis was performed with SPSS for Windows version 26.0, and the whole study protocol was approved by the institutional review board of Dankook University Hospital (approval No. 2020-11-015).

## 3. Results

### 3.1. Intrarater Reliability

Intrarater reliability is shown in Table 2. The kappa coefficients of all human raters showed almost perfect agreement except for Human 3 (a novice physician), who had only moderate agreement. The kappa coefficients of the model showed perfect agreement (intrarater kappa = 1.00), as expected. The PRRs of all human raters were above 90%. The NRRs of experienced human raters (Human 1 and Human 2) were above 90%, but Human 3 showed an NRR of only 68%. The PRR and NRR of the model were both 100%.

### 3.2. Interrater Reliability

The interrater kappa coefficients are shown in Table 3. All pairs of two human raters showed substantial agreement in both sessions, except that there was only moderate agreement between Human 2 and Human 3 in the second session. The machine and every human rater also showed substantial agreement in both sessions, except that there was only moderate agreement between the machine and Human 3 in the second session.

**Figure 3:**
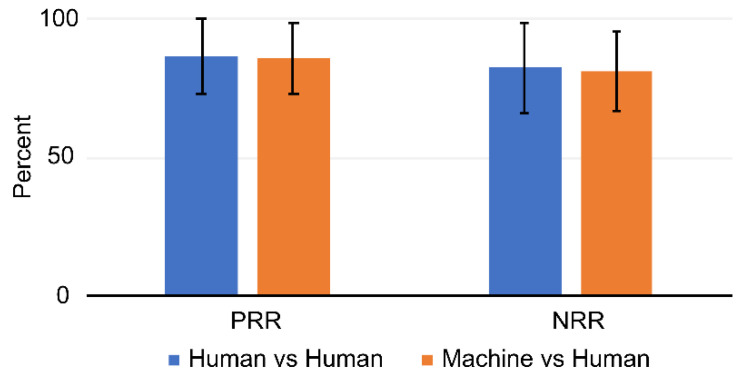
PRR and NRR values averaged between human raters and between machine and human raters.

The calculated PRRs and NRRs are shown in Table 4. Overall, the PRR values ranged from 62% to 100%, and the NRR values ranged from 50% to 100%. No particular pattern was found in the distribution of PRR or NRR among the human and machine ratings. The ratios were somewhat variable among the raters and between sessions. In order to delineate the difference in reliability, the PRR and NRR values were averaged and compared. The average PRR was 86.6% when measured between each pair of human raters and 85.5% when measured between the machine and each human rater. The average NRRs were 82.4%, and 81.3%, respectively. PRR and NRR values were not significantly different regardless of whether they were between human raters or between machine and human raters (Figure 3).

## 4. Discussion

One of the major limitations of VFSS is unsatisfactory interrater reliability. Its poor reliability may originate from the rapidity and complexity of the swallowing process and resultant difficulties in its analysis [25], as well as incomplete standardization of the definitions and judgment criteria of parameters [9]. Several methods have been used to improve the reliability of VFSS, including training and education [26], group discussion [25], directed search [27], frame-by-frame observation [5] and computer-assisted automated analysis [10,11,12,13,14,15]. Most previously proposed computer-assisted analyses use semiautomated algorithms that require human manual demarcation of salient anatomical structures [10,11,12,13,14,15]. To our knowledge, the deep learning model we proposed in our previous study was the first fully automated model capable of detecting laryngeal penetration or aspiration in VFSS images [20]. The model showed more than 90% accuracy, but its reliability has not been tested sufficiently. The reliability of computer-assisted analysis, whether with semiautomated or deep learning models, has never been compared with that of human examination. This is the first study designed to compare the reliability of machine and human examiners for VFSS analysis and demonstrate the reliability of VFSS analysis using a deep learning model.

Since there is not yet an absolute gold standard for the analysis of VFSS results, the significance of classical epidemiologic statistics, such as the kappa coefficient, intraclass correlation coefficient or positive and negative predictive values, may be limited for assessing the reliability or validity of VFSS analysis. Kuhlemeier et al. [8] proposed that the PRR and NRR, modified from the positive and negative predictive values, can be useful for verifying the reliability or agreement among raters in the absence of a gold standard. They used the PRR to denote the probability that a condition that has been judged to be abnormal by a rater will also be judged the same by a separate rater or in a second rating by the same rater [8]. Similarly, the NRR was used to denote the probability that a rating of ‘‘normal’’ would be followed by a second rating of ‘‘normal’’ either by a different rater or by the same rater at a different time [8]. In this study, we used the PRR and NRR in addition to the kappa coefficient to increase statistical strength.

The results of reliability analysis for VFSS data can be influenced by test videos because VFSS data frequently shows diverse findings according to the severity and type of dysphagia. If the test videos contain only mild or vague laryngeal penetrations and aspirations, raters may have difficulties in judgment, and the reliability will be lowered. If the videos contain only severe laryngeal penetrations and aspirations, agreement between the raters may appear excessively high because judgment of definite laryngeal penetration or aspiration might be easy for all raters. We made our best effort to include test videos with a balanced distribution of characteristics, including the gender and age of patients and the viscosity of the diet. Efforts were also made to include patients with diverse degrees of penetration and aspiration in the test dataset. In this way, we believe that selection bias was minimized in the measurement of reliability.

The experience of the raters may also affect the results of reliability analysis. [25]. Experienced raters usually have highly accurate standards of judgment, while less experienced raters can have confusion or difficulty in making decisions. We invited and compared three human raters with different levels of experience to minimize the effect of experience. The raters comprised one with more than 20 years of experience, one with approximately 10 years and one with approximately one year. We believe that the bias caused by different degrees of experience was minimized by comparing human raters with different experience levels. In addition to experiences, more extensive training also affected the difference between experienced and less experienced examiners because it had been recommended for precise use of the Penetration/Aspiration Scale [26].

As expected, the intrarater reliability was excellent for human and machine reading except in the novice physician (Human 3). Regarding interrater reliability, the kappa coefficients between the deep learning model and each human rater showed moderate to substantial agreement, except for Human 2 vs Human 3 and the machine vs Human 3 in the second session. Human 3 showed the lowest agreement with other human raters and machines as well as the lowest intrarater reliability, suggesting that experience may play an important role in the analysis of VFSS results by humans. It is reasonable to speculate that our deep learning model might be more reliable than an inexperienced human reader for VFSS analysis.

The PRRs showed inconsistent results both between human raters and between the machine and human raters, but the values were generally above 70%, except for Human 3 in the second session. It can be speculated that the agreement between experienced human raters and the deep learning model is high for positive results (the presence of penetration or aspiration). The lower PRR values between Human 3 and the other human raters as well as the machine may again suggest that interrater agreement may be affected by the raters’ experience level. The PRRs of the machine to the human raters showed almost perfect agreement (above 80%), although the PRRs of the human raters to the machine showed much lower values. The meaning of the difference between “machine-to-human” and “human-to-machine” PRRs is unclear. The NRRs, meaning the agreement for negative results (the absence of laryngeal penetration or aspiration), were generally lower, but not by a wide margin. To compare the agreement between the human raters and the agreement between the machine and human raters, we averaged and compared the PRRs and NRRs. The differences were not significant, suggesting that the overall agreement between the machine and human raters was noninferior to that between the human raters for both positive and negative results.

These results indicate that computer-assisted analysis using a deep learning model is a reliable method for detecting laryngeal penetration or aspiration through a VFSS. Considering its consistency and efficiency, deep learning computer analysis could provide good assistance to human examiners, who are vulnerable to fatigue and variability. It is anticipated that machine reading with a deep learning model will be able to improve the reliability and accuracy of VFSS analysis by reducing the time and effort required of human observers. The concept of computer-assisted detection of penetration or aspiration is of great clinical value for many reasons such as the potential for lower cost screening for aspiration or the facilitation of telehealth.

This study has several limitations. In the present study, human raters and the machine judged the existence of laryngeal penetration or aspiration only, although most VFSS examiners evaluate the depth and amount of laryngeal penetration or aspiration as well as its presence. The ultimate purpose of VFSS is not only to detect penetration or aspiration, but also to evaluate the pathophysiology and mechanism of swallowing. However, variables other than laryngeal penetration and aspiration were not considered in the analysis because the deep learning model was designed and trained only for the detection of laryngeal penetration or aspiration. Therefore, the machine described in this study is at best a prototype that proves that penetration/aspiration can be detected by computers, but in no way resembles human interpretation of VFSS at least for now. There was no distinction between penetration and aspiration in this study, although they have different clinical meanings [28]. Dynamics of continuous eating was not verified in this study because the analysis was limited to the video containing only one swallowing event. Additionally, the meaning and usefulness of the reliability results might be limited by the absence of a gold standard for comparison. For the same reason, selection bias could not be eliminated completely in choice of video files although we made every effort to avoid it. Despite these limitations, we believe that machine reading by a deep learning algorithm can assist human observers, helping to minimize the variability and improve the efficiency of VFSS analysis. Further studies are required to develop more sophisticated models that can assess VFSS images more comprehensively. The results presented in this study are only descriptive statistics. This study did not aim to determine the superiority or inferiority of machine reading, only to demonstrate its usefulness.

## 5. Conclusions

Computer analysis using a deep learning model can provide a reliable method for detecting the existence of laryngeal penetration or aspiration in VFSS images. This deep learning model has promising prospects for use in VFSS analysis although further research will be required to increase its reliability and accuracy.

## Figures and Tables

**Table 1:** Characteristics of VFSS dataset for test.

Factors		Number of Video Files (Number of Patients)	%
Gender	Male	87 (21)	50
	Female	86 (21)	50
Age (years)	40–49	35 (8)	20
	50–59	31 (7)	18
	60–69	30 (8)	17
	70–79	35 (7)	20
	80+	42 (12)	24
Viscosity of diet	Thick liquid	40	23
	Rice porridge	41	24
	Curd-type yogurt	35	20
	Thin liquid	33	19
	Cup drinking	24	14
Laryngeal penetration or aspiration	Absent	79	46
	PA2 2–3	44	25
	PAS 4–5	29	17
	PAS 6–8	21	12

**Table 2:** Intrarater reliability represented by kappa coefficients, PRR and NRR.

	Kappa	PRR (%)	NRR (%)
Human 1	0.830	93	91
Human 2	0.930	96	97
Human 3	0.693	98	68
Model	1.000	100	100

**Table 3:** The interrater Cohen’s kappa coefficients.

	Session	Human 2	Human 3	Machine
Human 1	1	0.672	0.781	0.660
	2	0.672	0.668	0.705
Human 2	1		0.672	0.732
	2		0.457	0.732
Human 3	1			0.705
	2			0.488

Scale for kappa coefficient: below 0.00 = poor agreement; 0.00–0.20 = slight agreement; 0.21–0.40 = fair agreement; 0.41–0.60 = moderate agreement; 0.61–0.80 = substantial agreement; 0.81–1.00 = almost perfect agreement.

**Table 4:** PRR and NRR values calculated between each human rater and the machine.

		PRR ^1^ (%)				NRR ^2^ (%)			
	Session	Human 1	Human 2	Human 3	Machine	Human 1	Human 2	Human 3	Machine
Human 1	1		73	91	73		100	88	99
	2		73	97	75		99	66	100
Human 2	1	100		99	86	70		70	87
	2	99		99	86	70		50	50
Human 3	1	92	73		75	85	99		100
	2	82	62		63	94	98		100
Machine	1	99	85	100		69	88	72	
	2	100	85	100		72	88	51	

^1^ positive reliability ratio = Abn(A and B)/Abn(A), ^2^ negative reliability ratio = Normal(A and B)/Normal(A).: A changes according to rows into Human 1, Human 2, Huma 3, Model, and B changes according to columns into Human 1, Human 2, Human 3, Model. See the method Section 2.3 for further details.

## Data Availability

The data presented in this study are available from the corresponding author upon reasonable request.
